# CASCADING IMPACT: LEVERAGING MEDICAL STUDENTS AS CATALYSTS FOR STOP THE BLEED TRAINING IN COMMUNITIES

**DOI:** 10.51894/001c.122876

**Published:** 2024-08-30

**Authors:** Elizabeth N. Montgomery, Bret Bielawski

**Affiliations:** 1 College of Osteopathic Medicine Michigan State University

## 27

### INTRODUCTION

Hemorrhage is the leading preventable cause of death from injury, yet saving a victim is possible if bystanders are equipped with necessary skills. Stop The Bleed (STB) was created by the U.S. Department of Defense in 2015 to accomplish this. Medical Students can register as STB Associate Instructors after attending any introductory bleeding course. To our knowledge, none of the seven medical schools in Michigan have a structured pathway to train medical Students in bleeding control and register them as STB instructors.

### OBJECTIVES

The primary objective is to demonstrate that Spartan Response, a more intense bleeding course, can train medical Students in hemorrhage control at Michigan State University College of Osteopathic Medicine (MSUCOM). The second objective is to assist medical Students with registering as STB instructors to increase the number in our community.

### METHODS

As a quality improvement initiative, I created Spartan Response. Tailored towards medical Students, it covers the anatomy and physiology of massive hemorrhage and emphasizes hands-on teaching of pressure application, bandaging, wound packing, tourniquet application and chest seal usage.

A QR code facilitates rapid sign-up for medical Students to become STB Instructors. Those who register can then be contacted later to assist with STB training in the community.

### RESULTS

Thus far, 247 MSUCOM first- and second-year medical Students, who were not previously registered as STB instructors, completed Spartan Response. Of those Students, 71 (28.7%) are now registered as Associate STB Instructors.

### CONCLUSION

Equipping medical Students with hemorrhage control skills and the confidence to employ them, enhances their emergency preparedness and creates a pool of STB Associate Instructors who can train more members of the community with STB. Assessing future community impact of the 71 instructors produced by Spartan Response, scalability suggests the potential training of 710 community members in a single STB training session in keeping with Stop the Bleed’s required 1:10 instructor-to-trainee ratio. Spartan Response can also be used at other medical schools in Michigan as a formal education tool, further increasing the number of responders in the state. Looking forward, we hope to demonstrate the number of community members trained by this approach.

